# Deciphering the core seed endo-bacteriome of the highland barley in Tibet plateau

**DOI:** 10.3389/fpls.2022.1041504

**Published:** 2022-10-28

**Authors:** Zhao Hao, Yanhong Wang, Xiaofang Guo, Ji De

**Affiliations:** School of Science, Tibet University, Lhasa, China

**Keywords:** highland barley, seed, endophytes, core endo-bacteriome, qingke

## Abstract

Highland barley (*Hordeum vulgare* var. *nudum* (L.) Hook.f., qingke) has unique physical and chemical properties and good potential for industrial applications. As the only crop that can be grown at high altitudes of 4200–4500 m, qingke is well adapted to extreme habitats at high altitudes. In this study, we analysed the seed bacterial community of 58 genotypes of qingke grown in different regions of Tibet, including qingke landraces, modern cultivars, and winter barley varieties, and characterised endophytic bacterial communities in seeds from different sources and the core endo-bacteriome of qingke. This study aim to provide a reference for the application of seed endophytes as biological inoculants for sustainable agricultural production and for considering microbe-plant interactions in breeding strategies. A total of 174 qingke seed samples from five main agricultural regions in Tibet were collected and subjected to investigation of endophytic endo-bacteriome using high-throughput sequencing and bioinformatics approaches. The phyla of endophytic bacteria in qingke seeds from different sources were similar; however, the relative proportions of each phylum were different. Different environmental conditions, growth strategies, and modern breeding processes have significantly changed the community structure of endophytic bacteria in seeds, among which the growth strategy has a greater impact on the diversity of endophytic bacteria in seeds. Seeds from different sources have conserved beneficial core endo-bacteriome. The core endo-bacteriome of qingke seeds dominated by Enterobacteriaceae may maintain qingke growth by promoting plant growth and assisting plants in resisting pests and diseases. This study reveals the core endo-bacteriome of qingke seeds and provides a basis for exploiting the endophytic endo-bacteriome of qingke seeds.

## Introduction

Endophytes are a class of non-pathogenic microorganisms that complete part or all their life cycles in plant tissues and are closely related to their host plants (Wilson, 1995; Farrar et al., 2014; Pitzschke, 2016). The existence of some endophytes can promote the growth of host plants and enhance the ability of host plants to utilise environmental nutrients, help plants resist biotic and abiotic stresses, and affect the competitiveness of host plants (Hardoim et al., 2012; Vandenkoornhuyse et al., 2015; Cope-Selby et al., 2017; Khalaf and Raizada, 2018; Tyc et al., 2020). Although complex endophyte communities are distributed in all plant organs, seed-distributed endophytes are of particular interest; in contrast, non-systemically horizontally transmitted endophytes, which systematically propagate through host plant seeds, and sexually vertically transmitted endophytes appear to have a higher probability of mutualism (Ewald, 1987; Rudgers et al., 2009; Saari et al., 2010; Gundel et al., 2011; Truyens et al., 2015).

Seeds are one of the most important stages in plant life. It is the beginning of a new life cycle of plants and the key to expanding the distribution range of plant populations and adapting to new environments (Truyens et al., 2015). Microorganisms in mature seeds are the endpoint of plant endophyte community assembly in seeds and the starting point of seedling endophyte community assembly, which may be an important source of endophytes in seedlings and adult plants (Khalaf and Raizada, 2018; Khalaf and Raizada, 2016). Compared to many soil-borne microorganisms, the endophyte community of seeds can better adapt to the life of plant tissues and has the advantage of colonisation (Truyens et al., 2015; Cope-Selby et al., 2017; Mukherjee et al., 2020). A suitable endophyte community play a positive role in the process of seed germination and seedling establishment, which can regulate the growth and development of seedlings and enhance the disease resistance (Pitzschke, 2016; Cope-Selby et al., 2017; Verma et al., 2017; Verma and White, 2018; Matsumoto et al., 2021). The development of biological inoculants with seed endophytes is considered a feasible method for improving agricultural productivity and developing sustainable agriculture; however, our research on seed endophytes is still limited (Mukherjee et al., 2021), particularly in plants grown in extreme environments (Compant et al., 2016).

The Qinghai-Tibet Plateau, known as the “Roof of the World” and the “Third Pole”, is the highest plateau in the world, with an average altitude of over 4,000 m and a changeable and extreme climate (Yao et al., 2012). The highland barley (*Hordeum vulgare* var. *nudum* (L.) Hook.f., qingke) is called “Qingke” in Chinese and “nas” in Tibetan. As one of the earliest crops domesticated by humans, qingke is an important crop for the production of food and medicines, beverages, and energy and has good industrial application potential (Palumbo et al., 2017; Zeng et al., 2018; Guo et al., 2020; Obadi et al., 2021; Yin et al., 2022). Qingke is the only crop that can be grown at high altitudes of 4200-4500 m. Its good frost resistance makes it a staple food for Tibetans at least 3500-4000 years ago, the main crop varieties accounting for 43% of the total area of ​​crops planted on the Qinghai-Tibet Plateau (Liu et al., 2013; d’Alpoim Guedes et al., 2015; Wang et al., 2017; Zeng et al., 2018). Because of the unique climatic conditions and geographical environment of the Qinghai-Tibet Plateau, compared with other barley genotypes, qingke had a founder effect event that lasted for 2,500 years before approximately 4,500-2,000 years, which makes qingke an untapped resource of unique seed endophytes (Zeng et al., 2018).

In this study, we employed a high-throughput sequencing approach to investigate the seed endophytic bacterial community in 58 qingke genotypes collected from five major agricultural regions in the Tibetan Plateau, including qingke landraces, modern cultivars, and winter barley. By analysing the diversity and composition of endophytic bacteria in these qingke seeds, the potential impact of seed core endo-bacteriome on the growth of qingke was inferred, which provides a basis for harnessing bacterial seed endophytes for sustainable barley production.

## Materials and methods

### Seeds sampling

The qingke landrace varieties were provided by the Tibet Academy of Agriculture and Animal Husbandry Sciences, and the modern cultivars and winter barley varieties were collected from Shigatse and Qamdo, Tibet, respectively. No pesticides were applied to the seed collection area. For comparison, we divided 58 qingke genotypes seeds from different sources into Lhasa landraces (Lh), Lhoka landraces (Lk), Nyingchi landraces (Ny), Qamdo landraces (Qa), Shigatse landraces (Sh), modern cultivars (MC), and winter barley (WB) 7 categories, and each seed contained three biological replicates for a total of 174 samples. One gram of barley seeds (composed18-22 seeds) was used for each sample.

### Total community DNA extraction

The seed samples were soaked in 75% alcohol for 5 min and then rinsed with sterile deionised water for 1 min. Surface cleaned seed samples are ground using a sterile mortar after liquid nitrogen freezing. Total DNA was extracted using a Plant DNA Extraction Mini Kit B (Mabio Co., Guangzhou, China) according to the manufacturer’s instructions under sterile conditions, and the purity and concentration of the total DNA were detected using a Thermo NanoDrop One spectrophotometre (Thermo Fisher Technology Co., USA).

### 16S rRNA gene amplification

According to the Earth Microbiome Project protocol (Thompson et al., 2017), universal bacterial primer set (pair) 515f (5′ GTGYCAGCMGCCGCGGTAA), 806r (5′ GGACTACHVGGTTWTAAT), and TaKaRa Premix Taq^®^ Version 2.0 (TaKaRa Biotechnology Co., Dalian, China) were used for each PCR amplification of the total DNA of the samples, and all PCR reactions were performed in triplicate. The PCR products were sent to Magigene Co., Ltd. (Guangzhou, China) to sequence the constructed amplicon library using the Illumina Nova PE250 platform after initial quality control and adapter ligation.

### Bioinformatics analysis

Clean paired-end reads were obtained after the primer sequences were removed using Cutadapt. Usearch V10 was used to filter unmatched tags to obtain the original spliced ​​sequence. Using Fastp V0.14.1 to trim the original spliced ​​sequence data with sliding window quality and obtain valid spliced ​​fragments, the UPARSE software was then used to cluster operable taxonomic units (OTUs) with 97% similarity. Representative sequences of each OTU were aligned in the SILVA V132 database using Usearch V10 to obtain species annotation information. After the annotation information was obtained, OTUs that could not be annotated to the kingdom and the OTUs annotated to chloroplasts, mitochondria, and archaea and their tags were deleted. Principal coordinate analysis (PCoA), ANOSIM analysis, and alpha diversity index were calculated using the vegan package of R V4.1.2, based on the flattened OTU table, and the Wilcoxon rank sum test and Kruskal-Wallis test were calculated using R V4.1.2. Correlation network analysis was performed using the Hmisc package of R V4.1.2, and Gephi was used for image drawing. Finally, we used LEfSe analysis to identify endophytic bacterial taxa with significantly different abundances in the seeds of different types.

## Result

### Diversity of endophytic bacteria in qingke seeds

**Figure 1:**
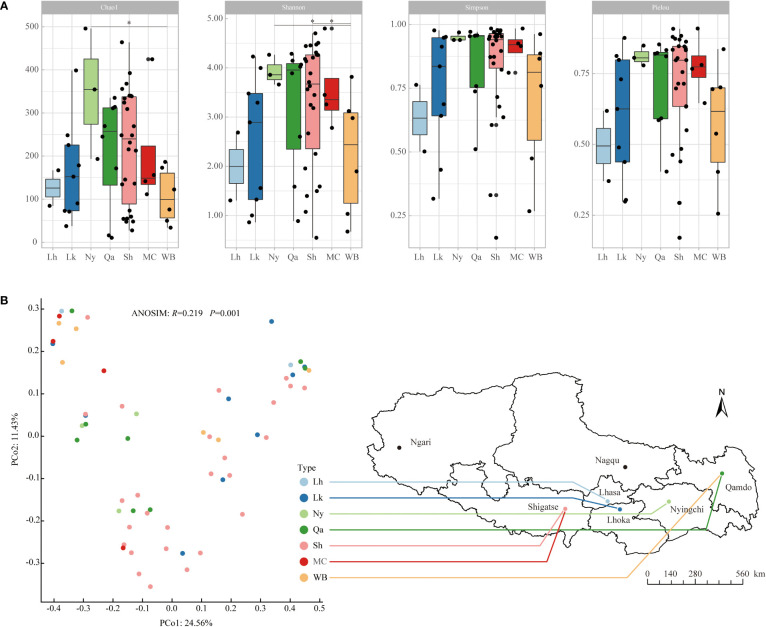
Alpha diversity and beta diversity of endophytic bacterial communities in different types of qingke seeds. **(A)** Alpha diversity. The “*” represent significant difference among different types of qingke seeds at 0.05 level. **(B)** Beta diversity was evaluated by PCoA ranking based on Bray-Curtis distance. Different colored dots represent different types of qingke seeds. Using ANOSIM analysis to assess the significance of differences in endophytic bacterial community structure in different types of qingke seeds. Lh, Lhasa landrace; Lk, Shannan landrace; Ny, Nyingchi landrace; Qa, Qamdo landrace; Sh, Shigatse landrace; MC, modern cultivars; WB, winter barley.

From the α diversity index (**Figure 1A**), in terms of the average Chao1 index, Nyingchi landraces were the highest, followed by Shigatse landraces, Qamdo landraces, modern cultivars, and winter barley is relatively low, the difference analysis showed that the Chao1 index of Nyingchi landrace was significantly higher than that of winter barley; in terms of the mean value of the Shannon diversity index, Nyingchi landrace was the highest, followed by modern cultivars, Shigatse landrace and Qamdo landrace were also higher, and Lhasa landrace was relatively lower, difference analysis showed that the Shannon diversity indices of Nyingchi and Shigatse landraces were significantly higher than those of winter barley; in terms of the average Simpson diversity index, Nyingchi landraces were the highest, followed by modern cultivars, Qamdo landraces and Shigatse landraces were also higher, and Lhasa landraces were relatively low. The uniformity of endophytic bacteria in qingke seeds from different types showed that the distribution of endophytic bacteria in Nyingchi landrace was the most uniform, the distribution of endophytic bacteria in modern cultivars, Qamdo landrace, and Shigatse landrace was also relatively uniform, and the distribution of endophytic bacteria in Lhasa landrace was the most uneven.

### Community structure of endophytic bacteria in qingke seeds from different sources

The β-diversity of qingke seed endophytes was assessed by PCoA analysis based on species-level Bray-Curtis distance, and the differences in the community structure of seed endophytes from different sources were calculated using ANOSIM (**Figure 1B**). The results showed significant differences in the community structure of endophytic bacteria in seeds from different sources (*R*=0.219, *P*=0.001). Among the qingke landraces, the endophytic bacterial community structures in seeds from Nyingchi and Qamdo were relatively similar, those from Lhoka and Shigatse were similar, and modern cultivars were similar to those of winter barley.

**Figure 2:**
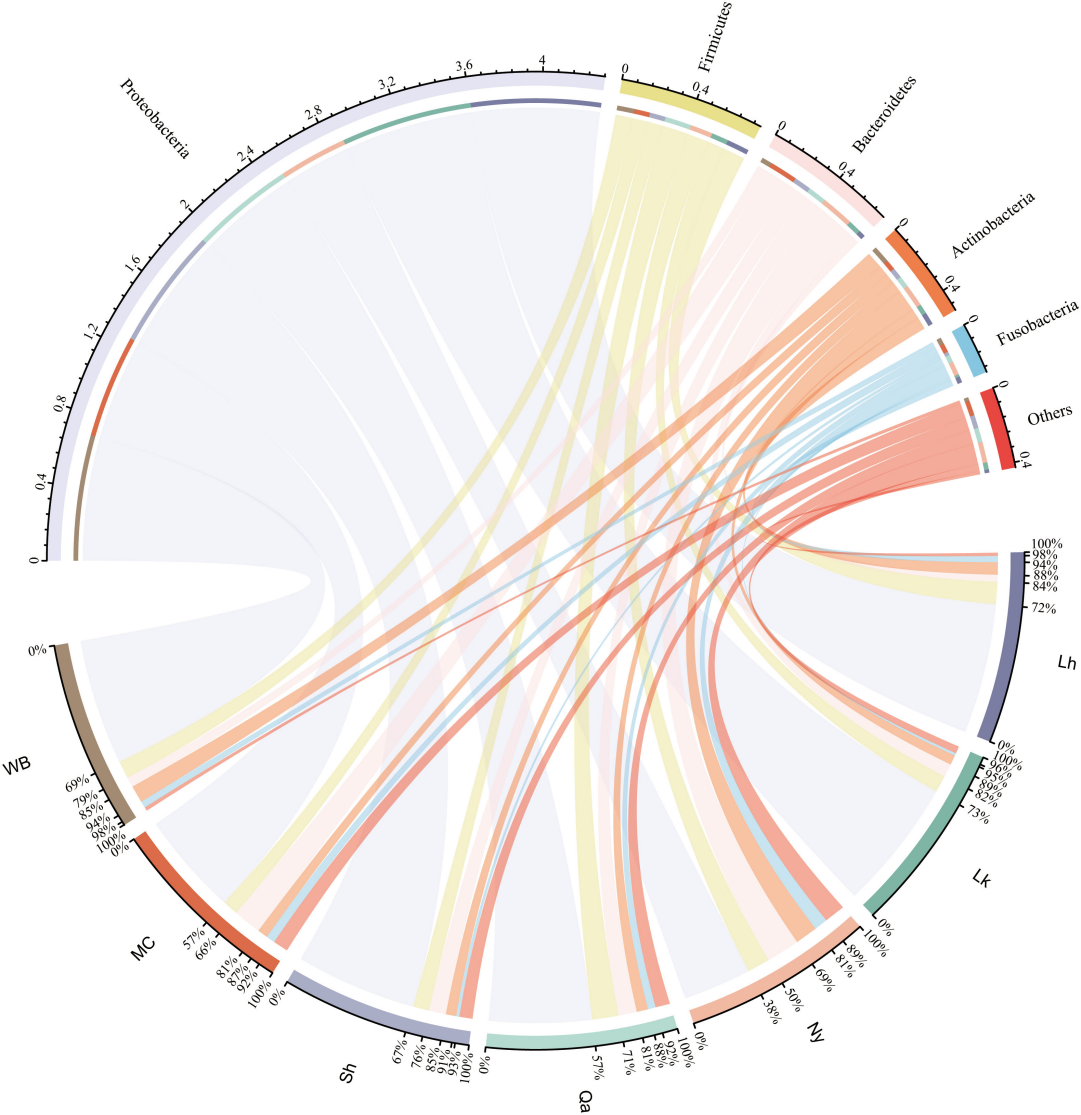
Composition of endophytic bacteria in different types of qingke seeds. Only the top 5 most abundant phyla are displayed, and the less abundant phyla are summarized as “Others”.

**Figure 3:**
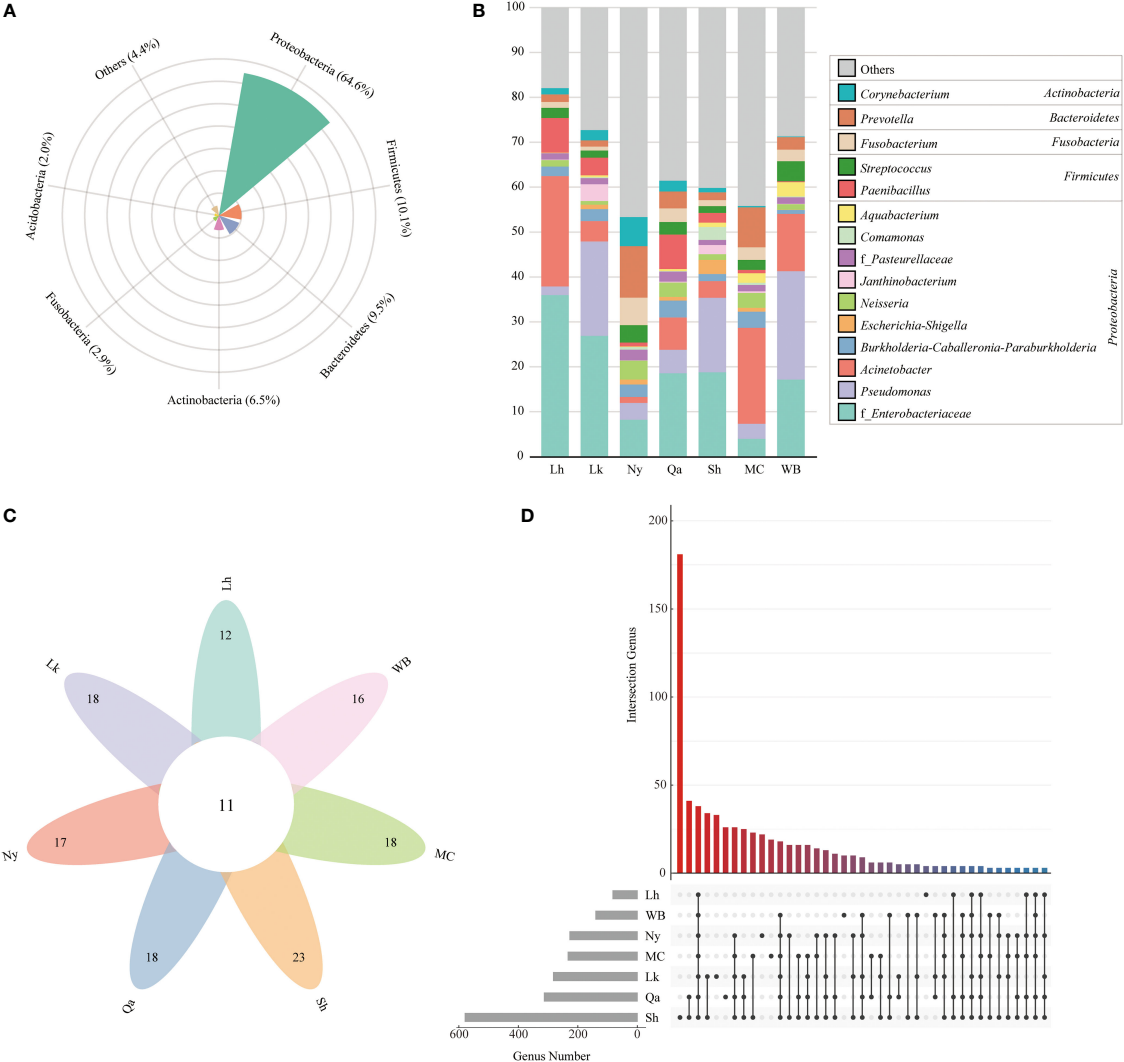
Similarity of endophytic bacterial composition of different types of qingke seeds. **(A)** The composition of endophytic bacteria in qingke seeds. The phyla with abundances below 2% are summarized as “Others”. **(B)** The genus composition of different types of qingke seeds. Taxa with abundances below 1% are summarized as “Others” **(C)** Venn diagram of the seed phylum level of different types of qingke. **(D)** The genus level upset of different types of qingke seed genus.

The composition of endophytic bacterial phyla in qingke seeds from different sources was similar. As shown in **Figure 2** and **Figure 3A**, Proteobacteria is the most important bacterial phylum (64.6%), in Lhasa landrace (71.7%), Lhoka landrace (72.6%), Nyingchi landrace (37.8%), Qamdo landrace (56.7%), Shigatse landrace (66.9%), modern cultivars (57.0%) and winter barley (69.5%) accounted for a higher proportion. Firmicutes (12.4%, 9.4%, 12.4%, 14.6%, 8.8%, 8.6%, and 9.7%), Bacteroidetes (3.8%, 7.3%, 18.4%, 10.2%, 9.5%, 15.6%, and 5.6%), the two accounted for 10.1% and 9.5% of the total OTU respectively. Actinobacteria (6.9%, 5.4%, 12.4%, 6.2%, 5.7%, 5.7%, and 9.5%), and Fusobacteria (3.4%, 1.3%, 7.4%, 4.3%, 1.8%, 4.9%, and 3.7%) accounted for a relatively small proportion, the two accounted for 6.5% and 2.9% of the total OTU number, respectively, and other categories accounted for less than 2% of the seeds from different sources. Among the 27 phyla, bacteria from 11 phyla were shared by all types of qingke (**Figure 3C**). Among the seeds from different types, Shigatse landrace had the most endophytic bacteria phyla, including 23 phyla, and landraces from Lhoka and Qamdo also had more endophytic bacterial phyla, including 18 phyla, which originated from the Lhasa landrace, and there were relatively few endophytic bacterial phyla, including 12 phyla.

The genus composition of qingke seeds from different sources showed (**Figure 3B**), Enterobacteriaceae in Lhasa landraces (36.0%), Lhoka landraces (26.9%), Nyingchi landraces (8.2%), Qamdo landraces (18.6%), Shigatse landraces (18.8), and winter barley (17.2) accounted for a higher proportion; *Acinetobacter* accounted for a higher proportion of Lhasa landraces (24.6%) and modern cultivars (21.4%); *Pseudomonas* accounted for Lhoka landraces (21.0%), Shigatse landraces (16.6%) and winter barley (24.1%) accounted for a higher proportion; *Prevotella* accounted for a higher proportion of Nyingchi landraces (11.5%) and modern cultivars (8.9%); *Paenibacillus* accounted for Qamdo landraces (7.7%), Lhasa landrace (7.7%) and Lhoka landrace (3.9%) accounted for a higher proportion. Among the seeds from all sources, Shigatse landraces had the most genera (579), Lhasa landraces had the least genera (82), and 38 genera (5.2%) were shared by different types of qingke seeds (**Figure 3D**).

### Correlation network of endophytic bacteria in qingke seeds

**Figure 4:**
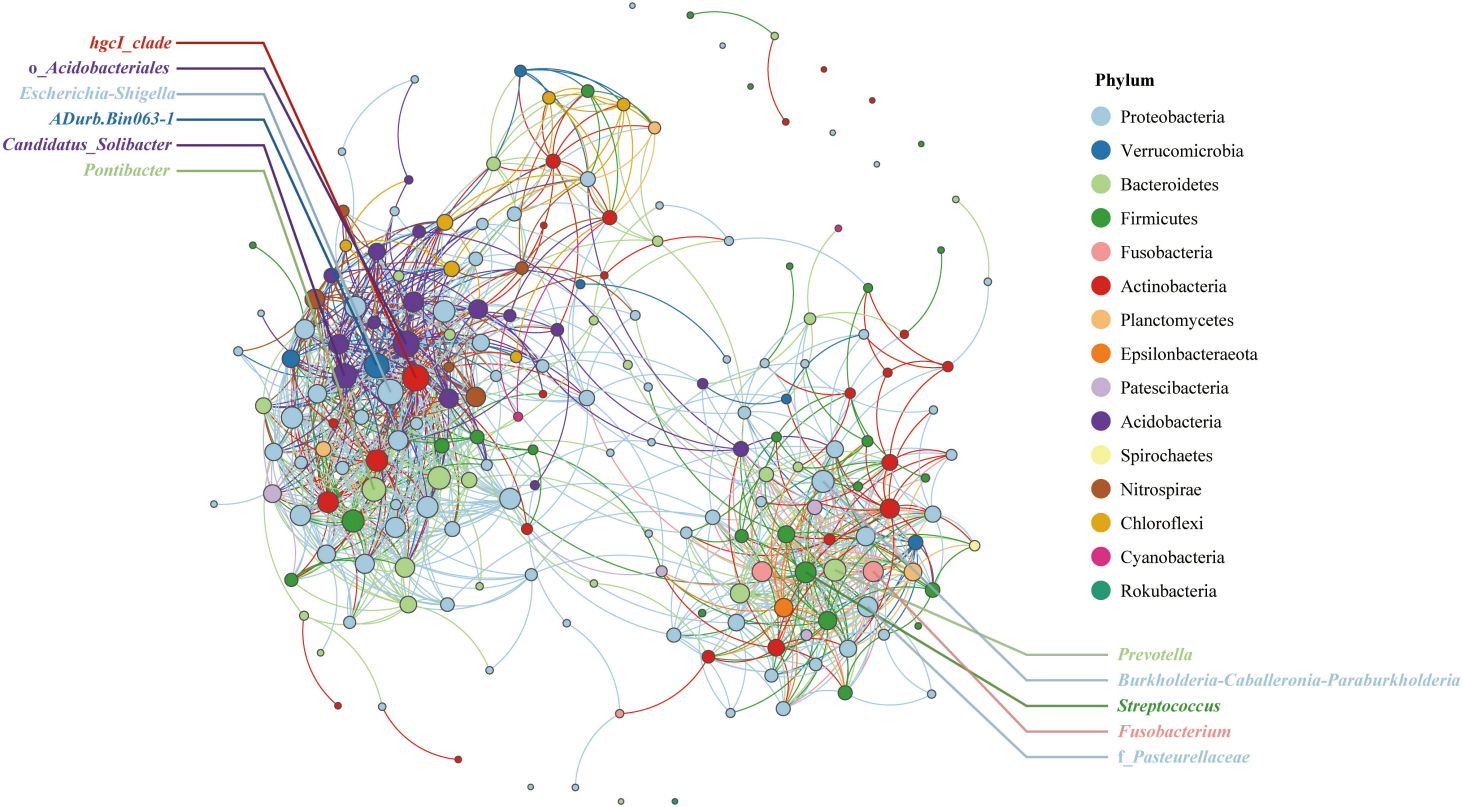
Correlation network analysis at the genus level of endophytic bacteria in qingke seeds. The node colors represent different bacterial phyla, and the node size is proportional to the degree.

Symbiotic bacterial network analysis can reveal the relationships among bacterial genera. To avoid complex images, the number of nodes is limited to 200. The network has a total of 1233 edges, of which 1193 edges are positively correlated and 40 are negatively correlated, and the average degree of the network is 12.3. As shown in **Figure 4**, the qingke endophytic bacterial genera can be divided into two groups. The *hgcI*_*clade* in the left-hand association group was closely related to other bacterial genera in the group, and its degree of connection was the highest among all nodes (2.1%). This was followed by o_Acidobacteriales (2.0%), *Escherichia*-*Shigella* (1.9%), *ADurb.Bin063*-*1* (1.8%), *CandidatusSolibacter* (1.7%) and *Pontibacter* (1.7%). In the right association group, *Prevotella* and *Burkholderia*-*Caballeronia*-*Paraburkholderia* were closely related to other bacterial genera, and the degree of connection was 1.4%. This was followed by *Streptococcus* (1.3%), *Fusobacterium* (1.2%), and f_Pasteurellaceae (1.2%).

### Differences in abundance of endophytic bacteria in qingke seeds from different types

**Figure 5:**
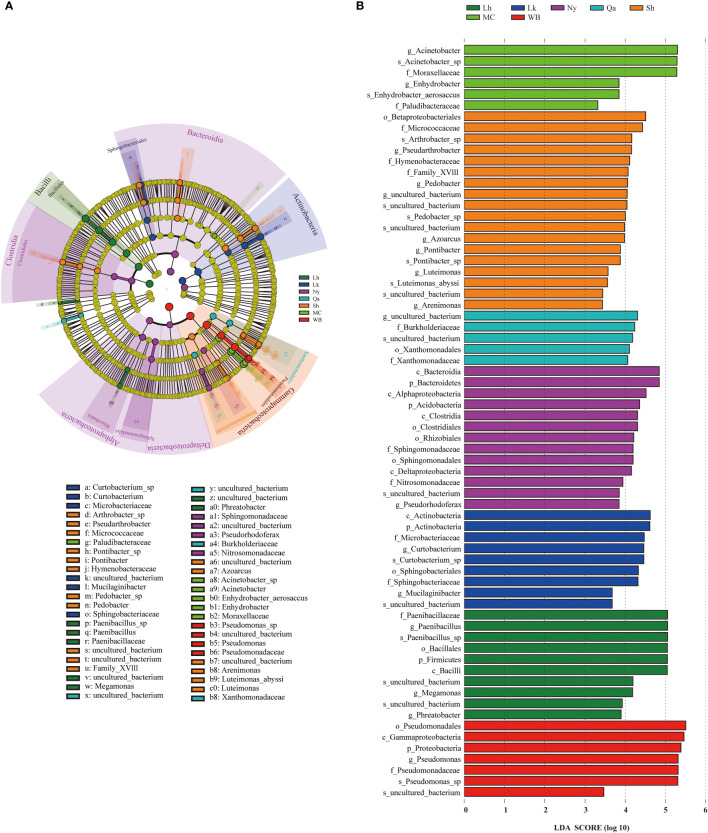
Using the Linear discriminant analysis Effect Size (LEfSe) method, the abundance of qingke seeds of different types of different bacterial taxa was revealed. **(A)** Clade diagram representation of the phylogenetic relationships of taxa with differences in abundance among qingke seeds from different sources. Nodes from inside to outside represent phyla, classes, orders, families, genera and species. **(B)** LEfSe analysis of endophytic bacteria in qingke seeds from different sources (*P* < 0.05, log LDA score threshold ≥ 3.3).

Significant differences in the endophytic bacterial communities or species in different types of qingke seeds were investigated using LEfSe. Statistical analysis from the phylum to species level was performed in the cladogram, and LEfSe confirmed an LDA score of 3.3 or higher (**Figures 5A, B**), and LEfSe analysis showed that there were 68 biomarkers. There were 10 taxa in Lhasa landraces, the taxa that contributed the most to Lhasa landraces were Paenibacillaceae (at the family level), *Paenibacillus* (at the genus level), and *Paenibacillus*_sp (at the species level). There are 9 taxa of Lhoka landraces, among which the taxa that contributes the most to Lhoka landraces is Actinobacteria (at the class level); there are 13 taxa of Nyingchi landraces, of which Bacteroidetes (at the phylum level) and Bacterodia (at the class level) are the taxa that contribute the most to Nyingchi landraces; there are 5 taxa of the landraces in Qamdo, of which the taxa that contributes the most to the landraces in Qamdo is the uncultured_bacterium (at the genus level) of the order Leptotrichaceae; There are 18 taxa of landraces in Shigatse, among which Betaproteobacteriales (at the order level) contributes the most to the landraces of Shigatse; there are 6 taxa of modern cultivars, of which the taxa that contributes the most to the modern cultivars is *Acinetobacter* (at the genus level); there are 7 taxa of winter barley, of which the taxa that contributes the most to winter barley is Pseudomonadales (at the order level).

## Discussion

Beneficial endophytes in seeds can promote seedling growth, increase the nutrient supply to host plants, and improve their resistance to biotic and abiotic stresses (Gond et al., 2015; Berg and Raaijmakers, 2018). At present, the possibility of targeted modification of seed microbiota to create optimal plant microbiota combinations to improve plant performance has been demonstrated (Mitter et al., 2017). The climatic conditions of the Qinghai-Tibet Plateau are harsh. As one of the most important crops on the Qinghai-Tibet Plateau, qingke has become a staple food for Tibetans because of its adaptation to extreme environments at high altitudes. Research on the endophytic bacteria in qingke seeds will provide a reference for the development and application of seed endophytes as biological inoculants in sustainable agriculture. In this study, we used high-throughput sequencing technology to study endophytic bacteria in the seeds of qingke landraces, winter barley, and modern cultivars widely planted in Tibet in five major agricultural regions. The average altitude of the five regions generally increased from east to west. Shigatse has the highest altitude, with an average altitude of more than 4000 m, followed by Lhoka (3700 m), Lhasa (3658 m), and Qamdo (3500 m), Nyingchi City has the lowest average altitude (3100 m). Alpha diversity showed that the abundance, diversity, and uniformity of endophytic bacteria in qingke seeds were the highest among the Nyingchi landraces, with the lowest altitude in the planting area and higher in the Qamdo area, where the planting area was relatively low. The climatic conditions in plateau areas are affected by altitude, compared with higher altitudes, areas with lower altitudes tend to have more suitable hydrothermal conditions. The more suitable hydrothermal conditions were possible reasons for the high diversity of endophytic bacteria in the seeds of qingke in the above two regions. It is worth noting that the abundance, diversity, and uniformity of endophytic bacteria in the landraces of Shigatse at the highest altitude in the planting area were higher than those of the Lhoka and Lhasa landraces, showing that the landraces of Shigatse have better adaptability to the high-altitude climate. As the main producing area of ​​qingke in Tibet, Shigatse has the largest number of qingke genotypes, the rich endophytic bacterial community of the landrace in Shigatse may play a role in the high-altitude adaptation of qingke. It is generally considered that microbial communities with higher diversity are more stable, similar to the findings of Abdullaeva et al. (2021), we found that the modern breeding process increased the diversity of endophytic bacteria in the seeds of qingke. Compared with the landraces, except Nyingchi, the diversity of endophytic bacteria in the seeds of the modern cultivars was higher. In addition, we found that the diversity of endophytic bacteria in the seeds of winter barley varieties was low. These results show that the seed endophyte community of modern cultivars has higher stability, modern breeding processes and different seed growth strategies have certain effects on the diversity of qingke seed endophytes, and plant growth strategy has a greater impact on seed endophytes.

The results of the PCoA and ANOSIM analyses showed significant differences in the endophytic bacterial community structure of qingke seeds from different sources. Owing to the founder effect event in the qingke population, our findings indicate that local planting for more than 2,000 years has significantly changed the endophytic bacterial community structure of qingke in different regions of Tibet. Among all landraces, the endophytic bacterial community structure of the Nyingchi and Qamdo landraces was relatively similar, and the endophytic bacterial community structures of the Lhoka and Shigatse landraces were relatively similar. The similar geographical location and altitude are possible reasons for their similar endophytic bacterial community structure.

Previous studies have shown that seed endophytes mainly belong to the phyla Proteobacteria and Firmicutes. Yang et al. (2017) also found that Proteobacteria and Firmicutes were the main endophytic phyla in the seeds of German barley cultivars. Rahman et al. (2018) studied barley seed endophytes in different geographical locations and harvest years in Germany and found that the relative abundance of Proteobacteria was the highest (94.8%), followed by Actinobacteria (3.4%), Firmicutes (1.2%), and Bacteroidetes (0.39%). Bziuk et al. (2021) studied the endophytes of seven different genotypes of barley seeds and found that Proteobacteria, Actinobacteria, and Firmicutes were the main endophytes in barley seeds. In our study, different types of qingke seeds had similar endophytic bacterial phyla composition: Proteobacteria (64.6%), Firmicutes (10.1%), Bacteroidetes (9.5%), and Actinobacteria (6.5%) were the main phyla of qingke endophytes. Proteobacteria, and Firmicutes, exhibiting biological control and plant growth-promoting traits in various plants and are the most important bacterial phyla in barley seeds (Rahman et al., 2018). In contrast to previous studies, Bacteroidetes and Firmicutes were relatively more abundant in our study. In studies of the rhizosphere microbiome of various plants, including barley, Bacteroidetes were found to be more abundant in the rhizosphere of various wild plant relatives than in domesticated species. In addition, wild relatives of domesticated barley have a higher abundance of Firmicutes (Pérez-Jaramillo et al., 2018). Studies have shown that endophytes originating from seeds can enter the soil and develop into the rhizosphere microbial community, suggesting that seed endophytes can shape the rhizosphere microbiota (Cope-Selby et al., 2017). For the connection between the seed endophytic microbiome and the rhizosphere microbiome, although this study did not examine the endophytic microbial community of the wild relatives of qingke, we suppose that compared with the previously studied barley genotypes, the endophytic microbiome of qingke seeds closer to wild barley populations, Bacteroidetes’ strong ability to degrade complex biopolymers, and organophosphorus mineralisation may help qingke obtain nutrients from the environment more easily (Lidbury et al., 2021).

In bacterial genus composition, although there are differences in endophytic bacteria in qingke seeds from different sources, at least 38 bacterial genera were conserved in the whole qingke population. In previous studies, we used to refer to the conserved microbiota as the core microbiota of the host plant, the vertically transmitted core microbiota in seeds is usually closely related to the healthy development and normal functional expression of the host plant (Johnston-Monje and Raizada, 2011). Consistent with the results of previous studies, we found that the core endo-bacteriome of Enterobacteriaceae accounted for a higher proportion of qingke seeds. Enterobacteriaceae are a group of gram-negative bacteria with strong degradation ability for organic compounds, which dominate in many plant endophytic microflora and are the endophytic core microflora of many plant seeds; members of this family are related to plant health and/or disease (Rossmann et al., 2012; Erlacher et al., 2015; Cernava et al., 2019; Zhang et al., 2020; Bziuk et al., 2021). In other relatively high-abundance core endo-bacteriome, *Acinetobacter* is a class of gram-negative bacteria, and some strains of this genus can grow hormones, solubilise phosphate, and produce siderophores, which can promote plant growth (Sachdev et al., 2010; Rokhbakhsh-Zamin et al., 2011; Adewoyin and Okoh, 2018). In this study, *Acinetobacter* accounted for a relatively high proportion of Lhasa landraces and modern cultivars and was the most abundant bacterial genus in the seeds of modern cultivars. Modern breeding activities may have changed the community structure of the seed endophytes to a certain extent (Johnston-Monje and Raizada, 2011). The plant growth-promoting effect of *Acinetobacter* strains is one of the possible reasons for the higher yield of modern cultivars. *Pseudomonas* is a class of Gram-negative bacteria with diverse species, different species of this genus exhibit diverse metabolic characteristics in different ecological niches, many species are closely related to plants and have excellent plant colonization ability, antibiotic production ability and Mineral phosphate solubilizing ability plays an important role in promoting plant growth and assisting plants in resisting pathogenic microorganisms (Shen et al., 2013; Oteino et al., 2015; Strunnikova et al., 2015; Girard et al., 2020). The genus *Pseudomonas* occupies a high proportion of Lhoka landraces, Shigatse landraces, and winter barley, and the enrichment of bacteria in this genus can reduce the diseases caused by pathogenic fungi in the soil during seed germination. Although most *Prevotella* species are derived from mammals, they are also found in paddy soils and in rice roots (Ueki et al., 2007). As a growth-promoting bacterium, *Prevotella* is the most important genus in Nyingchi landraces, which may be related to its higher optimum growth temperature of *Prevotella* (Arafat et al., 2020). Among all the regions involved in this study, Nyingchi had the lowest altitude and highest annual average temperature. Members of the genus *Paenibacillus* are biochemically and morphologically diverse, and many species of this genus are considered to promote plant growth (Xie et al., 2016). In this study, the *Paenibacillus* genus accounted for a higher proportion in the Qamdo, Lhasa, and Lhoka landraces, and the climate conditions and altitude of the above three regions were similar, and the relative abundance of *Paenibacillus* in seeds may be affected by climate and altitude. Podolich et al. (2015) suggested that plants have a core microbiome that responds dynamically to environmental factors, and our study verifies this conclusion.

Symbiotic bacterial network analysis divided the endophytic bacterial communities of Tibetan qingke seeds into two groups. The *hgcI*_*clade* is widely distributed in freshwater ecosystems worldwide; however, its role as a plant endophyte has not been investigated (Zhou et al., 2020). In another association group, *Prevotella* is a widely studied plant growth-promoting bacteria. Although *Burkholderia*-*Caballeronia*-*Paraburkholderia* is closely related to a variety of plants, its role in plants is unclear. Given that *Burkholderia*, *Caballeronia*, and *Paraburkholderia* have been reported to increase plant nutrient absorption, promote plant growth, and degrade complex organic matter (Madhaiyan et al., 2021), the effect of *Burkholderia*-*Caballeronia*-*Paraburkholderia* on qingke growth deserves further study.

In previous studies, it was found that the endophytic microbiota of plant seeds is affected by the plant growth environment, the endophytic microbiota of seeds produced under field conditions showed more activities related to the inhibition of plant pathogens, and the endophytic microbiota of seeds produced in the environment showed more activities related to promoting nutrient absorption (Bergna et al., 2018). Using LEfSe to analyse the endophytic bacterial community of qingke seeds from the phylum to the species level, it was found that the landraces in Shigatsehad the most different groups of endophytic bacteria, and the landraces in Qamdo showed the least difference. By analyzing the main differential endophytic bacterial groups of seeds from different types, it can be found that the main differential endophytic bacterial groups of Lhoka landrace and winter barley seeds can promote plant growth and resist pests and diseases (Palaniyandi et al., 2013). The main differential endophytic bacterial groups of Lhasa landrace seeds can promote plant growth (Arafat et al., 2020). The main differential endophytic bacterial groups in the seeds of Nyingchi landraces have the ability to degrade complex biopolymers (Clocchiatti et al., 2021). The main differential endophytic bacterial groups in the seeds of modern cultivars have the ability to promote plant growth and inorganic salt acquisition (Adewoyin and Okoh, 2018). The main differential endophytic bacterial groups of the seeds of Shigatse landraces is a common and important part of the plant microbiome, this bacterial family member interacts with arbuscular mycorrhizal fungi (AMF) and cooperates with AMF to participate in the adaptation process of plants to stress tolerance, increasing the nutrient acquisition and growth rate of plants (Emmett et al., 2021). The main differential endophytic bacterial taxa of the Qamdo landraces are members of a bacterial family (Leptotrichaceae) containing pathogenic bacteria, however, several studies have shown that some members of this family also have biological control traits (Lory, 2014; Das and Dhal, 2022). Although there were differences in the endophytic bacterial taxa of qingke seeds from different sources owing to climatic conditions, sowing strategies, and breeding processes, most of the major differential bacterial taxa had plant growth-promoting and biological control traits. It is worth noting that the main differential endophytic bacterial taxa of Shigatse landraces can interact with AMF, and AMF and seed endophytic microbiota have been shown to affect biotic or abiotic soil properties directly or indirectly, thereby affecting plant growth and communities (Idbella et al., 2021). The interaction between the qingke seed endophytic bacterial community and the soil microbiota may play a positive role in the establishment of qingke seedlings under harsh climatic conditions.

## Conclusion

In this study, high-throughput sequencing was used to study the endophytic bacteria of qingke seeds. In the analysis of different qingke landraces, modern cultivars, and winter barley, we found that: the modern breeding process has increased the endogenous bacterial diversity of qingke seeds, the growth strategy of winter barley overwintering reduced the diversity of endophytic bacteria, and there were significant differences in the bacterial community structure of different types of qingke seeds. In addition, we found that the core endo-bacteriome, dominated by Enterobacteriaceae, *Acinetobacter*, *Pseudomonas*, *Prevotella*, and *Paenibacillus*, is conserved in different types of qingke seeds and that the core endo-bacteriome may maintain qingke growth by promoting plant growth or assisting plants to resist pests and diseases. In the analysis of the association network of endophytic bacteria, a variety of key taxa of qingke endophytic bacteria, including *hgcI*_*clade*, *Prevotella*, and *Burkholderia*-*Caballeronia*-*Paraburkholderia*, were found. Further research on key taxa will help us better understand the endophytic bacterial communities of qingke seeds. This study reveals the community structure of the core endo-bacteriome of qingke seeds with multiple host-friendly traits and provides a feasible strategy for the application of qingke seed endophytes as bioinoculants for sustainable agricultural production of qingke and other high-altitude crops.

## Data availability statement

The data is presented in the study are both in the NCBI's Sequence Read Archive (SRA) repository, accession number PRJNA874323.

## Author contributions

XG, JD and ZH designed the study. YW, ZH, XG and JD performed the research. XG, JD, YW and ZH wrote the paper. All authors contributed to the article and approved the submitted version.

## Funding

This study was supported by National Key R&D Program of China (2021YFE0113700).

## Acknowledgments

We thank associate research fellow Da Wadunzhu (Tibet Academy of Agriculture and Animal Husbandry Sciences) and A.P Zhuo Ga (Tibet Agricultural and Animal Husbandry University) for providing barley seeds used in this study, prof. Chen Xiaoyulong (Guizhou University) and Magigene for their assistance in analysis of high-throughput sequencing data.

## Conflict of interest

The authors declare that the research was conducted in the absence of any commercial or financial relationships that could be construed as a potential conflict of interest.

## Publisher’s note

All claims expressed in this article are solely those of the authors and do not necessarily represent those of their affiliated organizations, or those of the publisher, the editors and the reviewers. Any product that may be evaluated in this article, or claim that may be made by its manufacturer, is not guaranteed or endorsed by the publisher.
